# Seroprevalence of Hepatitis A in Oman Prior to National Vaccination

**DOI:** 10.3390/jcm14196857

**Published:** 2025-09-28

**Authors:** Halima Al Shuaili, Mohamed El-Kassas, Khalid M. Al-Naamani, Zakaryia Al Muharrmi, Muhannad Al-Kobaisi

**Affiliations:** 1 Department of Internal Medicine, Division of Gastroenterology and Hepatology, The Medical City for Military and Security Services, Muscat 111, Oman; halima.alshuaili@gmail.com; 2 Endemic Medicine Department, Faculty of Medicine, Helwan University, Cairo 11795, Egypt; m_elkassas@yahoo.com; 3 Applied Science Research Center, Applied Science Private University, Amman 11937, Jordan; 4 Department of Microbiology and Immunology, Sultan Qaboos University Hospital, University Medical City, Muscat 123, Oman; muharrmi@squ.edu.om.com; 5 Microbiology and Immunology Department, College of Medicine and Health Sciences, Sultan Qaboos University, Muscat 123, Oman; alkobaisi@sfc-oman.com

**Keywords:** hepatitis A virus, hepatitis A antibodies, vaccination, seroprevalence, Oman

## Abstract

**Background:** The hepatitis A virus (HAV) is a major cause of acute viral hepatitis and a significant global health concern. This study provides a pre-vaccination baseline for Oman, enabling longitudinal comparison with post-hepatitis A vaccination cohorts. This study aimed to determine the pre-vaccination seroprevalence of HAV antibodies (anti-HAV) in Oman and explore the associated demographic factors. **Methods:** A cross-sectional study was conducted from April 2014 to August 2015 among patients attending the medical outpatient clinic of the Medical City Hospital for Military and Security Services. Demographic data were collected via a structured questionnaire, and serum samples were tested for anti-HAV immunoglobulin IgG and IgM using enzyme-linked immunosorbent assays. Multivariate analysis was performed to identify the predictors of anti-HAV seroprevalence. **Results:** Among 1975 participants, 88.1% were positive for anti-HAV IgG. The mean age was 37.4 ± 16.1 years; however, those negative for anti-HAV IgG were considerably younger (mean age: 24.8 ± 15.7 years). Anti-HAV IgG seroprevalence was 37% in individuals aged ≤18 years and 91% in those >18 years (*p* < 0.001). The factors associated with seropositivity included older age (*p* < 0.001), consuming food prepared outside the home (*p* < 0.001), occupation (*p* < 0.001), and education level (*p* = 0.003). In the multivariable analysis, only age showed a strong independent association with serostatus: per 10-year increase, the aOR for anti-HAV IgG seropositivity was 2.87 (95% CI 2.25–3.63; *p* < 0.001). **Conclusions:** Our study estimates show high anti-HAV IgG seroprevalence and serve as a pre-vaccination baseline for evaluating the hepatitis A vaccination program in Oman over time. Given the lower natural exposure among younger cohorts, continued routine vaccination, scheduled serosurveys, and strengthened surveillance are required to identify emerging immunity gaps and prevent future HAV outbreaks.

## 1. Introduction

Despite being preventable through vaccination, the hepatitis A virus (HAV) remains a significant global health burden, with an estimated incidence of 158 million cases in 2019, up from 139.4 million in 1990 [1,2,3]. In 2019 alone, the number of HAV-related deaths exceeded 39,000, highlighting the ongoing burden of the disease despite the availability of effective preventive measures [4]. The World Health Organization (WHO) has set a goal to eliminate viral hepatitis as a part of its global health sector strategies for 2022–2030, emphasizing the importance of vaccination, particularly in intermediate-endemic regions [5].

Transmission of HAV occurs primarily via the fecal–oral route through contaminated food, water, or direct person-to-person contact [6]. Its epidemiology varies based on socioeconomic factors, hygiene standards, and vaccination policies, leading to distinct regional patterns of transmission and immunity [1]. Endemicity is classified as high, intermediate, low, or very low based on the seroprevalence rates and age of exposure. In high-endemic regions, such as sub-Saharan Africa and South Asia, most infections occur in early childhood, are asymptomatic, and confer lifelong immunity, leading to a low adult disease burden [7,8,9]. In contrast, low-endemic regions, including North America, Western Europe, and parts of the Asia–Pacific, have declining childhood exposure, increasing susceptibility in adolescents and adults, who are more likely to experience symptomatic and severe disease [7,8,9].

Vaccination plays a critical role in controlling HAV transmission, with several countries implementing routine immunization programs. In Saudi Arabia, HAV seroprevalence among school-aged children declined from 52% in 1989 to 18.6% in 2008, reflecting the impact of improved hygiene and vaccination policies [10]. In 2017, surveillance data recorded 1175 laboratory-confirmed HAV cases in Oman, suggesting transmission of the virus 9. Several years later, a nationwide vaccination program was introduced for infants born after January 2019 [11]. Because our study preceded the hepatitis A vaccination policy, the present study represents a pre-hepatitis A vaccination baseline. Establishing these estimates as baseline values enables longitudinal comparison with post-vaccination cohorts and evaluation of the impact of vaccinations.

No comprehensive national seroprevalence study has been conducted in recent years, limiting the ability of policymakers to evaluate the effectiveness of current strategies or identify emerging immunity gaps in adolescents and adults. This is particularly concerning given Oman’s evolving demographic landscape, marked by rapid population growth, a substantial influx of expatriate workers, and increased regional and international mobility, all of which heighten the risk of HAV introduction and transmission. Furthermore, as natural immunity from past childhood exposure wanes in the context of improving hygiene, an increasing proportion of the population may remain susceptible into adulthood, when infection is more likely to cause severe disease and place a heavier burden on the healthcare system. The main aim of this study is to estimate the pre-vaccination HAV seroprevalence in Oman by age and risk groups, and to establish a baseline for assessing the post-2019 infant hepatitis A vaccination program, informing catch-up vaccination of adolescents and young adults, periodic serosurveys, and enhanced surveillance, consistent with the WHO 2030 goals.

## 2. Materials and Methods

### 2.1. Study Design and Setting

This cross-sectional study was conducted from April 2014 to August 2015 at the medical outpatient clinic of the Armed Forces Hospital, which later joined other medical establishments and was named the Medical City for Military and Security Services, Muscat, Oman. The current study data collection occurred before the initiation of hepatitis A vaccination in Oman in 2019; therefore, the estimates reflect baseline pre-vaccination immunity. Participants were recruited during routine outpatient clinic visits and provided informed consent before enrollment.

### 2.2. Study Population and Sampling

A total of 1975 individuals participated in the study. The eligibility criteria included patients attending the clinic for routine care or general consultations. Patients were excluded if they had previous hepatitis A vaccination. Convenience sampling was used to ensure a broad representation of different age groups, occupations, and socioeconomic backgrounds.

### 2.3. Data Collection

Demographic data were collected through a questionnaire administered by trained healthcare professionals. A structured questionnaire was reviewed by a panel of experts in infectious diseases and public health to ensure its content validity, and was pilot tested on a small group of patients to assess its clarity, relevance, and comprehension before full implementation. Information was gathered on age, gender, occupation, education level, travel history, primary source of drinking water, contact history, and frequency of consuming food prepared outside the home.

Venous blood samples were collected from all participants and tested for anti-HAV IgG and IgM using an enzyme-linked immunosorbent assay on the Roche Elecsys System analyzer (Cobas e 100, Roche Diagnostics, Mannheim, Germany). The assay was performed according to the manufacturer’s instructions in the in-house laboratory. Anti-HAV IgG positivity (equal to or more than 20 IU/L) was considered indicative of past HAV exposure or immunity, while anti-HAV IgM positivity was deemed to indicate an acute HAV infection. The assay’s sensitivity and specificity were 100% and 99.48%, respectively, according to the manufacturer [12].

### 2.4. Statistical Analysis

Descriptive statistics, including means, standard deviations, and proportions, are used to summarize the participants’ characteristics. Univariate analysis was performed to assess the differences in anti-HAV seroprevalence across demographic categories using chi-square tests for categorical variables and t-tests for continuous variables. A multivariate logistic regression model was used to identify independent predictors of anti-HAV seroprevalence. Given its role as a proxy for birth cohort and cumulative exposure, age was prespecified as a confounder and retained in all multivariable models irrespective of its statistical significance. For interpretability, age effects are also reported per 10-year increase. Additional covariates including occupation, education level, and frequency of eating food prepared outside the home were selected based on epidemiologic rationale and bivariable associations (screening threshold *p* < 0.20). Adjusted odds ratios (aORs) with 95% confidence intervals (CIs) are reported.

The data were analyzed using the Statistical Package for the Social Sciences (SPSS) software, version 22.0 (IBM Corp., Armonk, NY, USA).

### 2.5. Ethical Considerations

The study was approved by the institutional review board at MCMSS (approval number FMC-MEC 001/2014). Written informed consent was obtained from all participants prior to data collection. Confidentiality and anonymity were maintained throughout the study, and all laboratory procedures followed international ethical guidelines for human research.

## 3. Results

**Figure 1:**
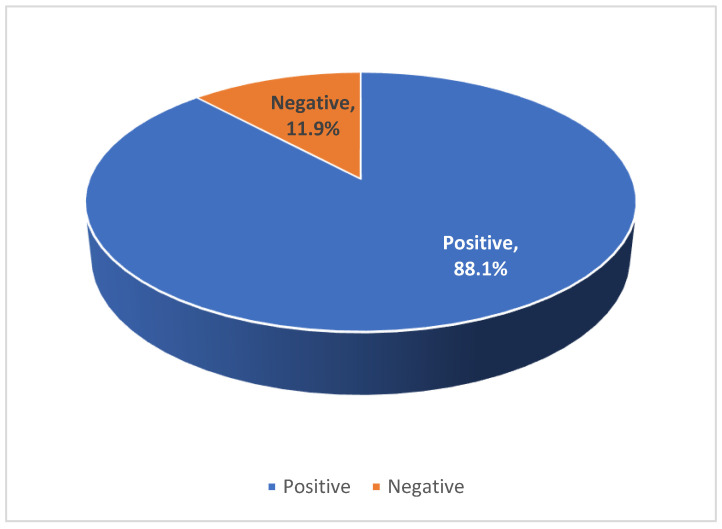
Seroprevalence of anti-HAV IgG positivity in the study population. (N = 1975). Abbreviations: anti-HAV: hepatitis A virus antibodies; IgG: immunoglobulin G.

A total of 1975 participants were included in the study: of these 63.5% were female, and the mean age was 37.4 ± 16.1 years. Overall, 1739 (88.1%) tested positive for anti-HAV IgG, while 236 (11.9%) were seronegative (Figure 1). None of the participants tested positive for anti-HAV IgM. Among individuals under 18 years of age, 50 (37.0%) were seropositive for HAV IgG. In contrast, 1689 adults (91.8%) tested positive for HAV IgG. This difference in seroprevalence was statistically significant (*p* < 0.001), indicating a marked disparity in immunity between the pediatric and adult populations (Table 1).

**Figure 2:**
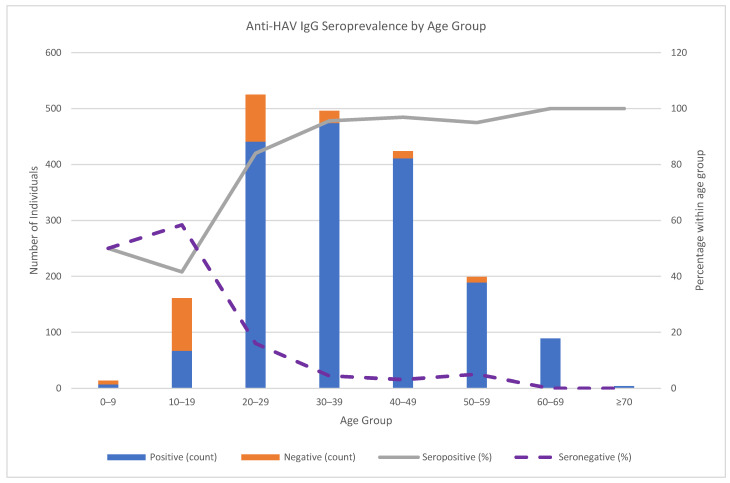
Age-stratified distribution of anti-HAV IgG seroprevalence in Oman. Stacked bars represent the number of seropositive (blue) and seronegative (orange) individuals within each age group. The solid gray line shows the proportion of seropositive individuals, while the dashed purple line represents the proportion of seronegative individuals.

The age-specific patterns demonstrated increasing Anti-HAV IgG seroprevalence with age. In the 0–9-year age group, Anti-HAV IgG positivity and negativity were each 50.0%; in the 10–19-year age group, 41.6% were positive, and 58.4% were negative. Positivity rose to 84.0% in the 20–29-year age group, 95.6% in the 30–39-year age group, 96.9% in the 40–49-year age group, 95.0% in the 50–59-year age group, and 100% in the ≥60-year age group (Figure 2).

The cohort included participants from various regions across Oman, with the highest representation from Al Batinah (34.0%), Muscat (24.4%), and Ad Dakhiliyah (22.9%). Other regions with notable representation included Al Sharqiya (10.1%), Ad Dhahirah (5.5%), and Dhofar (0.5%). Few participants originated from Al Buraimi (0.4%), Al Wusta (0.1%), or Musandam (0.1%).

The univariate analysis revealed that individuals who tested positive for anti-HAV IgG were significantly older than those who were seronegative (mean age: 39.13 ± 16.3 vs. 24.75 ± 15.7 years; *p* < 0.001, CI: 12.16–16.59). Anti-HAV IgG seroprevalence was comparable between genders, with no significant association between gender and anti-HAV IgG seroprevalence. However, occupation was significantly associated with anti-HAV IgG seroprevalence (*p* < 0.001), with office workers (92.1%) and non-office workers (93.5%) exhibiting higher seropositivity rates compared to unemployed individuals (88.6%) and full-time students (57.0%).

The educational level was also significantly associated with anti-HAV IgG seropositivity (*p* = 0.003), with the highest seroprevalence among individuals with no formal education (93.9%), followed by those with school-level education (86.9%), while university-educated participants had the lowest rate (84.8%). Travel history was not significantly associated with anti-HAV IgG seropositivity, indicating that international travel was not a major determinant of HAV exposure in this population. Similarly, the primary source of drinking water at the place of residence did not significantly impact the seroprevalence. However, the anti-HAV IgG seroprevalence was significantly associated with the frequency of consuming food prepared outside of the home, with lower seropositivity rates observed among participants who frequently ate outside (*p* < 0.001) (Table 2).

A multivariate binary logistic regression analysis was performed to identify the risk factors associated with HAV seroprevalence. Age was strongly associated with serostatus: per 10-year increase, the adjusted odds of seropositivity were 2.87 (95% CI 2.25–3.63; *p* < 0.001), indicating that younger individuals were less likely to be anti-HAV IgG seropositive. Dietary habits, occupation, and education level were not independently associated with HAV seropositivity in the multivariate model (Table 3).

## 4. Discussion

Hepatitis A remains a significant public health concern in Oman, with all cases systematically recorded by the Ministry of Health (MoH). Despite limited research on the annual incidence of HAV infections in the country, this study underscores the importance of identifying high-risk groups. The current study data were collected before the initiation of hepatitis A vaccination in Oman in 2019; therefore, these baseline data enable time-trend analyses (e.g., age-standardized seroprevalence, birth-cohort comparisons).

Our findings reveal a shifting epidemiological pattern in HAV seroprevalence within the Omani population. The overall anti-HAV IgG seroprevalence was 88.1% (Table 1/Figure 1), with a pronounced age gradient: 50.0% in ages 0–9, 41.6% in 10–19, rising to 96.9% in 40–49, and 100% in ≥60 years (Figure 2). In the multivariable analysis, age remained the dominant correlate (aOR for seropositivity 2.87 per 10-year increase; 95% CI 2.25–3.63; *p* < 0.001), indicating that susceptibility is high in adolescents and young adults. By contrast, sex showed no difference (Table 2). Occupation and education displayed crude differences (e.g., lower seropositivity among full-time students), but these attenuated after age adjustment, consistent with age/birth-cohort confounding. Age acts as a proxy for birth cohort and cumulative exposure, which is the main driver of anti-HAV IgG seropositivity in settings shifting from high to intermediate/low endemicity. Variables such as education and frequent eating outside the home are high among younger cohorts and therefore largely reflect age-related differences rather than independent effects. Moreover, anti-HAV IgG represents lifetime infection, whereas our covariates reflect current occupation/education/behaviors; this temporal mismatch plus likely non-differential measurement error reduces the magnitude of the adjusted effects. Given the high overall seroprevalence (88%), the statistical power to detect small residual effects is also limited. Collectively, these factors explain the attenuation of crude associations after age adjustment and are consistent with the epidemiology of HAV described by the WHO and global reviews [1,2,3,4]. Eating outside the home was associated in bivariable analyses yet was not independent after adjustment; travel history and primary water source were also not associated with anti-HAV IgG seropositivity.

The demographic shift observed in this study likely reflects improvements in sanitation, hygiene, and socioeconomic development over the past few decades, which have reduced natural childhood exposure to HAV [7,10], Additionally, increased access to clean water, urbanization, and changing household structures may have further limited transmission opportunities. While these trends represent public health successes, they paradoxically increase the pool of susceptible individuals in both adolescence and adulthood, where clinical manifestations of HAV are more severe [2,13].

The data suggest that individuals under 18 years of age are at highest risk for future HAV outbreaks, with those aged 19 to 29 years also displaying some degree of susceptibility. Although the per-year adjusted odds ratio (aOR) is modest, the decade-scale effect is substantial and concordant with the steep age gradient in the crude data. The age-related pattern, which showed low immunity in adolescents and young adults but near-universal immunity in older adults, indicates a potentially susceptible population. To translate this baseline into prevention, MoH as the leading health service provider in Oman should (i) sustain and continuously evaluate the current hepatitis A vaccination program initiated in 2019; (ii) implement targeted catch-up vaccination for school-age children and young adults (e.g., at school/college entry and other routine touch-points) similar to the catchup program for hepatitis B vaccination in Oman held between 2001 and 2005 [14]; (iii) offer risk-based vaccination to high-risk groups such as patients with chronic liver diseases, travelers to high-endemic areas, and groups involved in outbreaks; and (iv) strengthen case-based surveillance with rapid outbreak investigation. In addition to the previously mentioned steps, periodic serosurveys (focused on cohorts born after 2019) to monitor the hepatitis A vaccination program’s impact and detect immunity gaps early are important.

The Middle East and North African (MENA) region exhibits an intermediate level of HAV endemicity. While infection rates have declined in urban areas, they remain high in rural regions and other areas with special circumstances. Studies in the 1980s indicated nearly universal immunity in some of Oman’s neighboring countries such as Yemen [15]. However, by the 2000s, childhood immunity had significantly declined, as observed in countries such as Kuwait, Yemen, and the United Arab Emirates [4]. A study from Qatar, using surveillance data from the country’s largest tertiary hospital (2002–2006), reported 162 HAV cases in 2006 (incidence 1.9 per 10,000). Children <15 years accounted for 72.3% of cases, with incidence declining among Qataris but increasing among expatriates. These patterns align with the region’s epidemiologic transition, with reduced childhood exposure and a shift in susceptibility toward older ages and mobile populations [16].

While some countries in the region have implemented hepatitis A vaccination programs, others have not, increasing the risk of outbreaks among younger populations [7,17].

Vaccination programs and preventive measures are instrumental in reducing HAV incidence. A notable example is Saudi Arabia, where HAV incidence has decreased substantially over time. Between 1992 and 2003, the incidence rates fell from 14.0 to 9.0 per 100,000, with a further reduction to 8.0 per 100,000 by 2007 following an outbreak in 2004 [8]. In 2008, Saudi Arabia introduced a universal childhood vaccination program, utilizing inactivated HAV vaccines as a two-dose schedule at 18 and 24 months of age. This initiative led to a significant reduction in incidence, from 8.02 to 2.54 per 100,000 by 2010—a 90% decrease in total cases. However, a slight resurgence in HAV cases was observed from 2016 to 2018, affecting adults aged 15–44 and >45 years due to gaps in vaccination coverage [8,18].

HAV is primarily transmitted via the fecal–oral route, with risk factors varying across populations. These determinants are largely influenced by sanitation standards, socioeconomic conditions, age, travel history, maternal educational level, and, to a lesser extent, occupational exposure [19,20,21,22,23]. Although the majority of studies have reported no significant association between occupational status and HAV seroprevalence, some observed a lower prevalence among students compared to workers [24,25]. This discrepancy may be attributed to age-related differences, as students are typically younger and therefore have had less cumulative exposure to HAV over time. In regions undergoing epidemiological transition from high to intermediate endemicity, reduced immunity among young adults and students is more plausibly linked to improved hygiene practices during childhood rather than occupational exposure [21]. Supporting this, large-scale outbreak investigations in the United States between 2016 and 2019 revealed that only approximately 3.8% of HAV cases occurred among food handlers, and among those, 67% had identifiable high-risk factors such as homelessness status [25,26]. Collectively, these findings underscore that the risk of HAV infection is chiefly shaped by environmental, socioeconomic, and demographic variables.

Given our finding of high HAV immunity among older adults in Oman compared with lower rates in younger generations, the cost–benefit of serological testing before vaccination requires careful consideration. Since the national HAV vaccination program only began in 2019, younger cohorts remain the main susceptible group. In this context, routine serology in older adults may help avoid unnecessary vaccination costs, whereas direct vaccination of younger individuals is likely more cost-effective. This approach is also consistent with the Centers for Disease Control and Prevention (CDC), which does not recommend testing for HAV antibodies prior to vaccination [27]. Tailoring vaccination strategies by age group could therefore optimize both resource allocation and public health impact.

This study is the first to examine HAV seroprevalence trends in Oman, revealing a significant shift in epidemiology. Our findings indicate low immunity among younger populations, heightening their risk for outbreaks. Although Oman implemented a national vaccination program for newborns in 2019, further research is needed to assess its impact on HAV transmission and immunity trends. These findings provide a critical baseline prior to the introduction of the HAV vaccination program in 2019. Future serosurveys are essential to evaluate the program’s effectiveness, identify immunity gaps, and inform targeted interventions to reduce the HAV burden in Oman.

Strengthening surveillance systems, expanding vaccination coverage, and implementing targeted public health interventions are essential for effective HAV control and outbreak prevention. However, the current study has some limitations that need to be acknowledged. The participants were limited to individuals attending outpatient clinic for routine care or general consultations. As a result, this may introduce selection bias, as certain groups of the general population such as school children and adolescents may have been under-represented. This could affect the external validity and generalizability of the study findings. Due to its cross-sectional design, exposures and outcomes were assessed at the same time; so, causal relationships and temporal ordering cannot be determined. Additionally, the temporal gap between the current study data collection and the implementation of hepatitis A vaccination requires updated surveillance. Given Oman’s transition toward lower early-life exposure and higher susceptibility at older ages, we regard the <18-year findings as hypothesis-generating and call for nationally representative school- or household-based serosurveys to evaluate the post-2019 hepatitis A vaccination program’s impact, detect immunity gaps for targeted catch-up vaccination, and enhance immunization efforts for at-risk groups.

## 5. Conclusions

The current study provides a pre-vaccination baseline for hepatitis A immunity in Oman. As routine hepatitis A vaccination was introduced for children born ≥2019, future surveillance should include post-2019 birth cohorts through periodic serosurveys and strengthened case-based reporting to evaluate the hepatitis A vaccination program’s impact. The age-specific profile, characterized by low immunity in adolescents and young adults, indicates a susceptible group. Therefore, vaccination policy should prioritize targeted catch-up vaccination for school-age children and young adults, enhanced monitoring of adolescent cohorts, and risk-based vaccination for high-risk adults, alongside ongoing infant vaccination and public health education. Due to the under-representation of children and adolescents in this study, these subgroup estimates are hypothesis-generating and require confirmation in nationally representative pediatric and adolescent surveys.

## Figures and Tables

**Table 1:** Distribution of anti-HAV IgG seroprevalence according to age group (N = 1975).

Anti-HAV IgG Status	Age Group, n (%)		*p* Value
	<18 Years (n = 135)	>18 Years (n = 1840)	
Positive	50 (37.0)	1689 (91.8)	<0.001
Negative	85 (63.0)	151 (8.2)	

Abbreviations: anti-HAV: hepatitis A virus antibodies; IgG: immunoglobulin G.

**Table 2:** Factors associated with anti-HAV IgG seroprevalence in the study population (N = 1975).

Characteristic	Anti-HAV IgG Status, n (%)			*p* Value
	Total	Positive (n = 1739)	Negative (n = 236)	
Gender				
Male	714 (36.2)	631 (88.4)	83 (11.6)	0.740
Female	1254 (63.5)	1102 (87.9)	152 (12.1)	
Age (years)				
Mean ± SD	37.4 ± 16.1	39.1 ± 16.3	24.8 ± 15.7	<0.001 *
Occupation				
Office work	315 (15.9)	290 (92.1)	25 (7.9)	<0.001 *
Field work	417 (21.1)	390 (93.5)	27 (6.5)	
Full-time student	86 (4.4)	49 (57.0)	37 (43.0)	
Unemployed	961 (48.7)	851 (88.6)	110 (11.4)	
Education level				
No formal education	247 (12.5)	232 (93.9)	15 (6.1)	0.003 *
School	855 (43.3)	743 (86.9)	112 (13.1)	
University	323 (16.4)	274 (84.8)	49 (15.2)	
History of travel abroad				
Yes	659 (33.4)	589 (89.4)	70 (10.6)	0.225
No	1173 (59.4)	1026 (87.5)	147 (12.5)	
Primary source of drinking water				
Piped	1063 (53.8)	941 (88.5)	122 (11.5)	0.253
Bottled	584 (29.6)	505 (86.5)	79 (13.5)	
Well	283 (14.3)	255 (90.1)	28 (9.9)	
Consumption of food prepared outside of the home				
>3 times/week	240 (12.2)	197 (82.1)	43 (17.9)	<0.001 *
1–2 times/week	317 (16.2)	268 (84.5)	49 (15.5)	
Rarely	1369 (69.3)	1231 (89.9)	138 (10.1)	

Abbreviations: anti-HAV: hepatitis A virus antibodies; IgG: immunoglobulin G; SD: standard deviation. * Statistically significant at *p* < 0.05.

**Table 3:** Independent predictors of anti-HAV IgG seroprevalence.

Predictor	B	OR ^†^	95% CI		*p* Value
			Lower	Upper	
Age	−0.105	0.9	0.879	0.922	<0.001 *
Occupation					
Office work	Ref *	-	-	-	-
Field work	−0.474	0.623	0.323	1.201	0.157
Full-time student	0.674	1.963	0.952	4.048	0.068
Unemployed	0.138	1.148	0.66	1.998	0.624
Education level					
No formal education	Ref *	-	-	-	-
School	0.114	1.121	0.552	2.276	0.752
University	0.088	1.092	0.521	2.289	0.817
Consumption of food prepared outside of the home					
>3 times/week	Ref *	-	-	-	-
1–2 times/week	−0.353	0.702	0.371	1.328	0.277
Rarely	−0.242	0.785	0.467	1.321	0.362

Abbreviations: anti-HAV: hepatitis A virus antibodies; IgG: immunoglobulin G; OR: odds ratio; CI: confidence interval. * Ref (reference) = the baseline category for that variable in the logistic regression. **^†^** OR = This OR is per year; the per-10-year aOR is ≈ 2.87 (95% CI ≈ 2.25–3.63).

## Data Availability

All data generated or analyzed during this study are included in this article. Further inquiries can be directed to the corresponding author.
